# Targeting notch pathway enhances rapamycin antitumor activity in pancreas cancers through PTEN phosphorylation

**DOI:** 10.1186/1476-4598-10-138

**Published:** 2011-11-10

**Authors:** Kevin Vo, Barushi Amarasinghe, Kay Washington, Adriana Gonzalez, Jordan Berlin, Thao P Dang

**Affiliations:** 1 College of Pharmacy, The University of Tennessee, Memphis, TN, USA; 2 Division of Hematology and Medical Oncology, Department of Medicine, Vanderbilt Medical Center, Nashville, TN, USA; 3 Department of Pathology, Vanderbilt Medical Center, Nashville, TN, USA; 4 Division of Hematology and Medical Oncology, Department of Medicine, University of Virginia, Charlottesville, VA, USA

## Abstract

**Background:**

Pancreas cancer is one of most aggressive human cancers with the survival rate for patients with metastatic pancreas cancer at 5-6 months. The poor survival demonstrates a clear need for better target identification, drug development and new therapeutic strategies. Recent discoveries have shown that the role for Notch pathway is important in both development and cancer. Its contribution to oncogenesis also involves crosstalks with other growth factor pathways, such as Akt and its modulator, PTEN. The mounting evidence supporting a role for Notch in cancer promotion and survival suggests that targeting this pathway alone or in combination with other therapeutics represents a promising therapeutic strategy.

**Results:**

Using a pancreas cancer tissue microarray, we noted that Jagged1, Notch3 and Notch4 are overexpressed in pancreas tumors (26%, 84% and 31% respectively), whereas Notch1 is expressed in blood vessels. While there was no correlation between Notch receptor expression and survival, stage or tumor grade, Notch3 was associated with Jagged1 and EGFR expression, suggesting a unique relationship between Notch3 and Jagged1. Inhibition of the Notch pathway genetically and with gamma-secretase inhibitor (GSI) resulted in tumor suppression and enhanced cell death. The observed anti-tumor activity appeared to be through Akt and modulation of PTEN phosphorylation. We discovered that transcriptional regulation of RhoA by Notch is important for PTEN phosphorylation. Finally, the mTOR inhibitor Rapamycin enhanced the effect of GSI on RhoA expression, resulting in down regulation of phospho-Akt and increased *in vitro *tumor cytotoxity.

**Conclusions:**

Notch pathway plays an important role in maintaining pancreas tumor phenotype. Targeting this pathway represents a reasonable strategy for the treatment of pancreas cancers. Notch modulates the Akt pathway through regulation of PTEN phosphorylation, an observation that has not been made previously. Furthermore, we discovered that this regulation is dependent on RhoA/Rock1 activation. Enhanced phospho-Akt suppression when GSI is combined with rapamycin suggests that targeting both pathways will lead to a greater efficacy in the treatment of patients with pancreas cancer.

## Background

The Notch pathway is an evolutionarily conserved pathway important for cell fate determination in development as well as in cancer. In development, Notch is involved in tissue patterning and morphogenesis through cell differentiation, proliferation and apoptosis. The Notch family in mammals consists of four receptors (Notch1-4) and five ligands (Jagged1, 2 and Delta-like 1, -3, -4). In the canonical pathway, Notch receptors are activated by membrane-bound ligands, resulting in several intramembrane proteolytic cleavages that untether the cytoplasmic domain (NICD) from the cytoplasmic membrane. The NICD translocates to the nucleus and activates the transcription of target genes, such as those belonging to the *Hairy/enhancer of split *and *Hairy/enhancer of split-related with YRPW motif *families [1]. In cancer, Notch crosstalks with numerous oncogenic pathways, such as Akt, TGF-β and src signaling [2-4]. In certain context, the interaction between Notch and other oncogenic pathway is independent of the canonical HEY and HES activation [5].

While accounting for only 4% of estimated new cases of cancer in both men and women, pancreas cancer is the fourth leading cause of cancer-related death in the United States [6]. The median survival for patients with advanced pancreas cancer remains at 5-6 months, a rate that has not changed significantly over the last decade [7]. Thus, identification of new targets is needed to improve clinical outcome. Current literature suggests that Notch pathway plays an instrumental role in pancreas cancer. In the developing pancreas, Notch regulates the ratio between the exocrine and endocrine cell mass, supporting its role in controlling cell-fate determination [8]. RT-PCR showed that Notch pathway components were overexpressed in a small set of pancreas tumors. Furthermore, activated Notch cooperates with TGF-β in the expansion of undifferentiated precursor cells and in the promotion of PanIN progression to anaplastic pancreas cancer [9,10].

In this study, we examined the prevalence of Notch receptors and ligands in a large number of patients with pancreas cancers. Using immunohistochemistry (IHC) on a tissue array, we discovered that Notch3 was most often overexpressed in pancreas cancer, followed by Notch4. Conversely, Notch1 was expressed in the vasculature within the tumor mass but not in malignant cells. Furthermore, inhibiting Notch activation reduced tumor phenotypes and Akt phosphorylation in pancreas cancer. While previous studies have shown that Notch-dependent activation of Akt is a result of transcriptional downregulation of PTEN, we noted that in our system, Notch regulated PTEN phosphorylation but not PTEN expression. Our results show that this regulation is dependent on RhoA and Rock1, an observation that has not been previously described. Finally, rapamycin, an inhibitor of the mTOR pathway, greatly enhanced Notch-dependent inhibition of Akt and tumor cytoxicity *in vitro*. This effect appears to be dependent of RhoA. Taken together, our observations further support a role for Notch in pancreas cancer and suggest a new strategy in targeting pancreas cancer.

## Results and Discussion

### Notch Receptors and Ligands Are Expressed in Resected Pancreas Cancer

**Figure 1:**
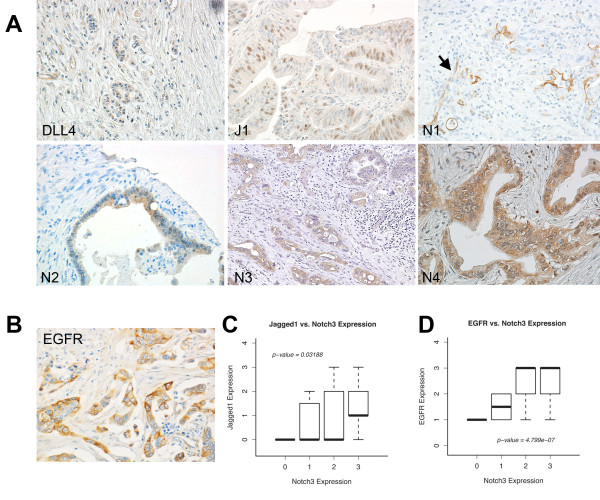
**Expression of Notch Receptors and Ligands in Pancreas Cancer**. (A) Representative examples of some pancreas tumors expressing Delta-like 4 (DLL4), Jagged1(J1), Notch3 (N2), Notch3 (N3), and Notch4 (N4) by IHC. No tumor expressed Notch1 (N1), but its expression can be detected in blood vessels (arrow). (B) A representative example of a pancreas tumor expressing high level of EGFR. While the expression of Notch ligands and receptors does not correlate with clinical characteristics, Notch3 expression correlates positively with that of Jagged1 (C) and that of EGFR (D).

**Figure 2:**
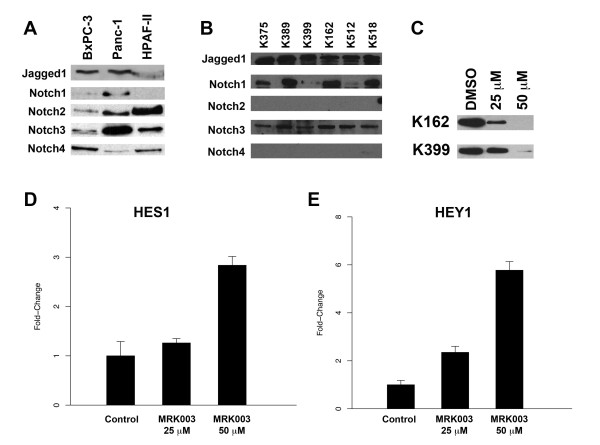
**Notch Receptors are Expressed in Pancreas Cancer Cell Lines, and Their Inhibition Is Inde-pendent of The Canonical Pathway**. (A) As opposed to resected pancreas cancers, expression of all four NICDS or activated forms of the Notch receptors can be seen in the human pancreas cancer cell lines. Both BxPC-3 and Panc-1 cell lines also express Jagged1. (B) Murine pancreas cell lines, developed from mice overexpressing K-rasG12D and TGF-β knockout, express only Notch1 ICD and Notch3 ICD. There was no appreciable difference in Notch expression K162, K152, and K518. (C) Inhibition of Notch3 using the γ-secretase inhibitor MRK003 resulted in the loss of activated Notch3. (D, E) However, enhanced transcription of Notch-dependent genes HES1 and HEY1 determined with RT-PCR suggests that the effect of Notch inhibition on pancreas cancer cells is not through the Notch canonical pathway.

The prevalence in expression of a potential oncogene helps determine the significance of its role in cancer. To better understand the role of Notch pathway in pancreas cancer, we developed a pancreas tissue microarray with associated clinical data from 86 patients (Table 1). We also examined the expression of Notch1-4 and their ligands, Jagged1 and DLL4. Notch3 was most prevalent with greater expression in 84% of resected cancers, followed by Notch4 at 31% (Table 2 Figure 1). Interestingly, none of the tumor cells expressed Notch1, and only one of the 86 tumors surveyed expressed Notch2. Notch1 and DLL4 were expressed predominantly in endothelial cells, suggesting that, while not significantly expressed in tumor cells, they are important in tumor angiogenesis. We also tested the dataset for correlation between different Notch family members and clinical characteristics, such as overall survival, stage and tumor grade. No association between Notch receptors and clinical characteristics was observed. However, we noted that Notch3 expression correlated with Jagged1, but not for Delta-like 4, suggesting that Jagged1 is the ligand for Notch3 (Figure 1C, Table 3) [11]. Of note, eighty-five percent of the tumors surveyed with IHC exhibited high expression of EGFR (Figure 1B). Notch3 also correlates with EGFR expression (Figure 1D, Table 4), consistent with our previous finding in lung cancer that Notch3 and EGFR pathways cooperate in maintaining the oncogenic phenotype [12]. Notch receptors are activated by proteolytic cleavages after ligand binding, resulting in the release of the cytoplasmic domain (NICD). We were able to demonstrate that several human pancreas cancer cell lines expressed the activated forms or NICD of Notch receptors (Figure 2A). In addition, pancreas cancer cell lines developed from overexpressing K-rasG12D and TGF-β knockout mice showed Notch1 ICD and Notch3 ICD expression (Figure 2B), further supporting the role of Notch pathway in pancreas cancers [13]. Similar to our previous observation, Jagged1 is also highly expressed in nearly all of cell lines tested [14]. We found no difference in Notch expression between cell lines with K-ras mutation alone (K162, K512, K518) and those with both K-rasG12D and TGF-β knockout (K375, K389, K399). When K162 and K399 were treated with MRK003, γ-secretase inhibitor, dose-dependent down regulation of activated Notch3 was observed (Figure 2C). Interestingly, while we observed suppression of the activated form of Notch, we observed a rise in HES1 and HEY1 transcripts, suggesting that Notch modulates cancer phenotype in pancreas through non-canonical pathways (Figure 2D, E).

**Table 1:** Patients' Characteristics

		n (%)
**Age**	Median	66
	Range	37-84
	< 65	39 (45)
	≥ 65	38 (44)

**Gender**	Male	48 (56)
	Female	38 (44)

**Race**	White	81 (94)
	Black	2 (2)
	Unkown	3 (3)

**Tumor Size**	≤ 1 cm	4 (5)
	> 1 and ≤ 2 cm	21 (25)
	> 2 and ≤ 3 cm	30 (36)
	> 3 cm	28 (34)

**pT**	T1	1 (1)
	T2	7 (8)
	T3	75 (88)
	T4	1 (1)

**pN**	Yes	46 (54)
	No	39 (46)

**pM**	Yes	5 (6)
	No	81 (94)

**Stage**	IA & IB	3 (3)
	IIA	33 (38)
	IIB	44 (51)
	III	1 (1)
	IV	5 (6)

**Tumor Grade**	1	17 (20)
	2	34 (40)
	3	35 (41)

**Chemotherapy**	Yes	30 (35)
	No	32 (37)
	Unknown	24 (28)

**Pos-Op XRT**	Yes	21 (24)
	No	39 (45)
	Unknown	26 (30)

**Table 2:** Expression of Notch receptors and ligands in resected pancreas cancer

Grade	DLL4	Jagged1	Notch3	Notch4
n	71	73	75	70
1	2	4	9	6
2	1	8	24	8
3	6	7	30	8

Total	9	19	63	22

% Positivity	13	26	84	31

**Table 3:** Pearson's Correlation between Jagged and Notch 3

	Notch3				
Jagged1	0	1	2	3	Total
**0**	4	5	18	6	33
**1**	0	1	7	12	20
**2**	0	2	8	7	17
**3**	0	0	1	1	2
**Total**	4	8	34	26	72

p-value = 0.01039

**Table 4:** Pearson's Correlation between EGFR and Notch3

	Notch3				
EGFR	0	1	2	3	Total
**1**	3	4	3	1	11
**2**	0	4	12	7	23
**3**	0	0	20	19	39
**Total**	3	8	35	27	73

p-value = 4.799e-07

### Inhibiting Notch Activation Reduces Malignant Phenotype and Induces Apoptosis

**Figure 3:**
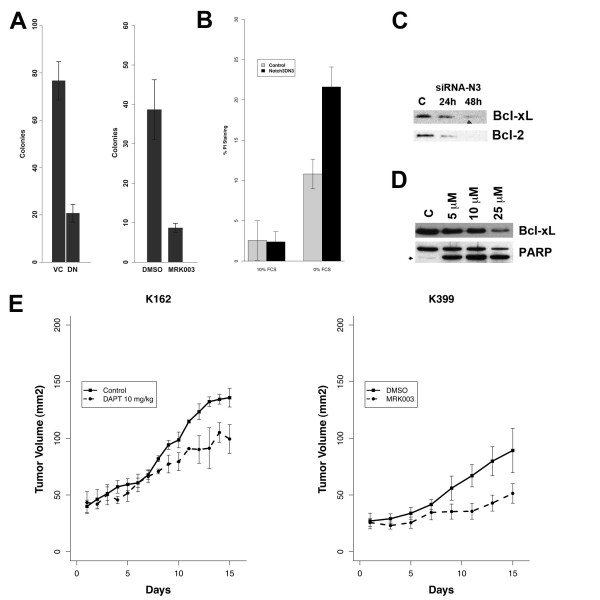
**Inhibition of Notch Pathway Results in the Loss of Tumor Phenotype and a Decrease in Proliferation**. (A) Inhibition of Notch signaling pathway using the dominant-negative Notch3 receptor (DN) and MRK003 markedly reduces the size of the colonies formed in soft agar of pancreas cancer cell line BxPC3, compared with control. (B) Similar to our previous observation in lung cancer, inhibition of Notch pathway using the DN receptor enhances cancer cells dependency on exogenous growth factor. Cells transfected with the DN construct shows a higher death rate in 0% serum, measured using PI staining, compared to control and cells grew in 10% fetal calf serum (FCS). (C). Using siRNA, loss of Notch3 reduces expression of Bcl-2 family proteins such as Bcl-xL and Bcl-2 in BxPC3. (D) A similar result is obtained when the γ-secretase inhibitor MRK003 is used. Loss of Bcl-xL also coincides with the increase in cleaved PARP, a measure of apoptosis. (E) Both γ-secretase inhibitors DAPT (10 mg/kg i.p.) and MRK003 (100 mg/kg, given through oral gavage in 3 consecutive days/week) attenuate tumor growth in K399 and K162 subcutaneous xenograft models.

To determine whether inhibiting Notch activation reduces tumor phenotype, we utilized both dominant-negative Notch3 receptor and a γ-secretase inhibitor (GSI). When BxPc3 was transfected with dominant-negative Notch3 or treated with 25 μM of MRK003, colonies were significantly reduced in number, as compared to vector controls (VC) or DMSO control (C) (Figure 3A). A significant body of literature has supported a role for Notch signaling in apoptosis. Similar to our previous observation in lung cancer, inhibiting Notch in serum-free condition resulted in enhanced cancer cell death measured with PI staining (Figure 3B). The Bcl-2 family plays an important role in apoptosis through the activation of the mitochrondria-dependent caspase pathway. Using Notch3 siRNA, we showed that Notch regulates Bcl-xL expression and Bcl-2 (Figure 3C). When MRK003 was used, a similar effect on Bcl-xL could be found, accompanied by an increase in cleaved PARP, a marker of caspases activation (Figure 3D). To determine whether γ-secretase inhibitors possess activity *in vivo*, we inoculated xenografts with K162 and K399 cell lines developed from a mouse model of pancreas cancer. The γ-secretase inhibitors DAPT and MRK003 suppressed tumor growth by 25% to 50%, suggesting that the Notch pathway plays a role in the survival of cancer cells in both *in vitro *and *in vivo *models (Figure 3E).

### GSI Inhibits Akt Activation and PTEN Phosphorylation

**Figure 4:**
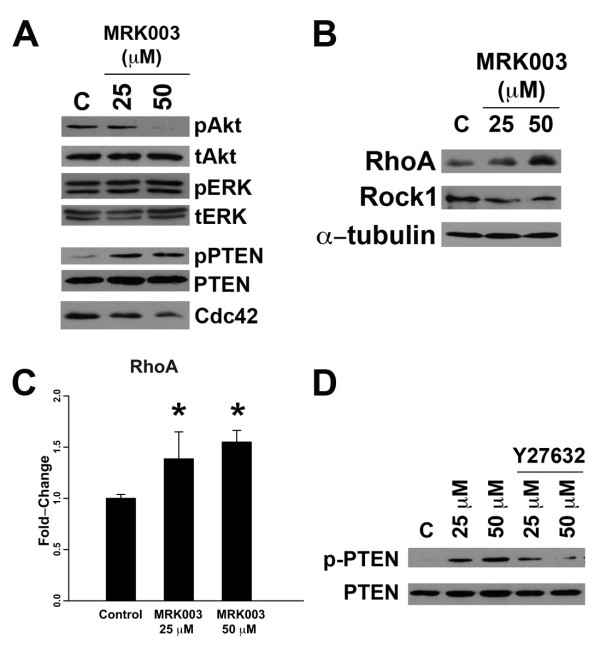
**Notch Inhibition Results in Downregulation of Akt Activation Through Rho-Dependent PTEN phosphorylation**. (A) Notch inhibition with MRK003 led to a decrease in Akt phosphorylation, but not ERK phosphorylation in K399. While the level of PTEN did not change with MRK003 treatment, expression of pPTEN was enhanced. Cdc42, a down-stream target of PTEN was also reduced. (B) MRK003 induced expression of the PTEN regulator, RhoA, but not Rock1. (C) Transcription of RhoA was induced 1.5× with MRK003, suggesting that the induction of RhoA by Notch inhibition is transcriptionally mediated. (*) denotes statistical significance compared with control. (D) This induction of phospho-PTEN was ameliorated in the presence of Rock1 inhibitor Y27632 at 30 μM (lanes 4 and 5), suggesting that Notch's effect on phosphoPTEN depends on the Rock/Rho pathway.

The Notch pathway is known to crosstalk with other oncogenic pathways such as the EGFR and the Akt pathway [12,15]. Interestingly, unlike observations in lung cancer, inhibition of the Notch pathway in pancreas cancer had no appreciable effect on ERK activation (Figure 4A). On the other hand, Akt phosphorylation was inhibited by MRK003 in pancreas cancer cell line K399. PTEN (phosphatase and tensin homolog) is a well-known negative regulator of Akt. In hypoxia, Notch1 has been shown to suppress PTEN transcription, leading to Akt activation [15]. However, while Notch is known to regulate Akt through the transcriptional regulation of PTEN, we did not detect a difference in total PTEN levels. Rather the phosphorylation of PTEN at Ser380 was altered, when GSI was used (Figure 4A). While not much is known about the phosphorylation of PTEN, recent evidence suggests that it regulates protein stability [16]. While some findings indicate that phosphorylation of PTEN improves stability but reduces PTEN function, others have shown that the loss of phospho-PTEN in migrating cells leads to the activation of Akt [17]. Cdc42, a member of the Rho GTPase family, is important in Akt-mediated cell survival and motility, and its activation is inhibited by PTEN [18,19]. We noted a decrease in Cdc42 when treated with GSI, suggesting that Notch regulates Akt-dependent cell survival through PTEN and Cdc42.

How PTEN is regulated through phosphorylation is intensely investigated. In a recent model of chemotaxis proposed by Li *et al*., Rock1, a member of the Rho-associated, coiled-coil containing protein kinases, is activated by Rho-GEF (guanine nucleotide-exchange factor) and RhoA, another Rho GTPase family member. Activated Rock1 then binds and phosphorylates PTEN [17,20]. Rho proteins and Rock proteins are important regulators of cell migration, proliferation and apoptosis [21]. To examine the role of the Rho GTPase pathway in Notch-induced PTEN phosphorylation in pancreas cancer, we examined the effect of GSI on Rock1 and RhoA. Interestingly, we noted an increase in the expression of RhoA with increasing dose of GSI, whereas the expression of Rock1 remained essentially unchanged (Figure 4B). The effect of Notch signaling on RhoA appears to be transcriptionally mediated (4C). To determine whether Notch modulation of PTEN phosphorylation is dependent on RhoA/Rock1, we examined the effect of GSI in the presence of Rock1 inhibitor Y27632 (4D). Whether the observations in the chemotaxis model can be translated into a cancer model requires further validation. The loss of PTEN phosphorylation by GSI in the presence of Y27632 suggests, however, that the Notch effect on PTEN depends on the RhoA/Rock1 pathway.

### Rapamycin Enhances GSI Antitumor Activity Through the Regulation of Akt

**Figure 5:**
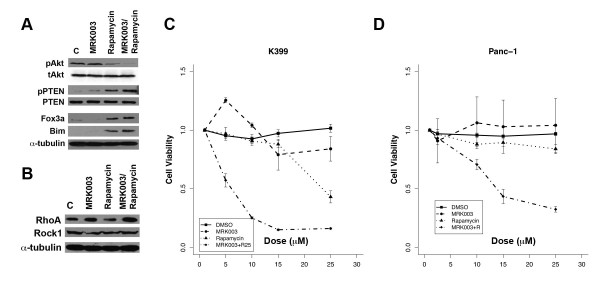
**Notch Modulates Akt Activation and Cooperates with mTOR Inhibitor in Tumor Proliferation**. (A) Akt inactivation and PTEN phosphorylation by Rapamycin (10 μM) are enhanced when MRK003 (10 μM) is added. MRK003 also enhances induction of pro-apoptic Fox3a and Bim by the mTOR inhibitor rapamycin. (B) Notch inhibition results in higher expression of RhoA, and the induction of RhoA is enhanced when rapamycin was combined with MRK003. No change is observed with Rock1. (C, D) In the MTT survival assays, the combination of MRK003 and rapamycin also leads to higher tumor cytoxicity *in vitro*, compared to either agents alone, in both murine K399 and human Panc-1 pancreas cancer cell lines.

The observed redundancy in oncogenic pathways may require that multiple pathways are inhibited in order to enhance tumor cytotoxicity. The PI3K/Akt/mTOR pathway is activated in the majority of pancreas cancers. Because of the crosstalk between Notch and Akt, we examined whether the combination of the mTOR inhibitor Rapamycin and MRK003 will result in improved tumor cytotoxicity. While some studies suggest that Rapamycin induces Akt activation, we noted that in K399 rapamycin inhibits Akt phosphorylation, and that this inhibition was enhanced, when Rapamycin was combined with MRK003 [22] (Figure 5A). Again, we observed a change in phospho-PTEN, but not total PTEN, when Notch pathway is inhibited. Furthermore, the level of phospho-PTEN was increased when MRK003 was combined with rapamycin. Foxo3a is a member of the forkhead family which acts as tumor suppressor by promoting cell cycle arrest and apoptosis. It is inactivated by Akt. The combination of Rapamycin and MRK003 led to a slight increase in the tumor suppressor Foxo3a and pro-apoptotic Bim, a member of the BH-3 only Bcl-2 family. Moreover, we noted an increased expression of RhoA, when cancer cells were treated with MRK003, and the change was enhanced when Rapamycin was added (Figure 5B). No change in Rock1 level was detected. Taken together, these observations support the hypothesis that Notch and mTOR cooperate in regulating Akt through PTEN phosphorylation and RhoA.

### Notch Inhibition Enhanced Rapamycin-dependent Growth Suppression in pancreas Cancer Cells

While results from preclinical studies using mTOR inhibitors in pancreas cancers have been promising, their low efficacy in early clinical studies indicate that these agents possess minimal clinical activity when administered as single agents [23]. Redundancy in the biological system and results from clinical trials suggest that targeting multiple targets will result in augmented tumor suppression. Because we observed Akt suppression when GSI was added to Rapamycin, we tested whether inhibiting the Notch pathway will enhance tumor suppression with mTOR inhibitor *in vitro*. In both human and murine pancreas cell lines, K399 and Panc-1, respectively, the combination of MRK003 and rapamycin inhibited proliferation to a greater degree than Rapamycin or MRK003 alone (Figure 5C, D). These findings suggest that Notch can enhance Rapamycin in inhibiting pancreas cancer growth through the modulation of Akt.

## Conclusions

Overexpression of Notch receptors and ligands in pancreas cancer supports the hypothesis that this developmental pathway plays an important role in this type of cancer. However, the lack of correlation between Notch pathway compounds, clinical characteristics and outcome does not support their use as biomarkers.We observed that Notch3 is expressed in cancer cells, whereas Notch1 is mainly expressed in blood vessels. Differences in expression pattern among the various Notch pathway components suggest a non-redundancy in functions. We hypothesize that in cancer Notch3 is important for tumor survival, whereas Notch1 mediates the response to hypoxia through the regulation of angiogenesis. This hypothesis is supported by previous observations from other investigators [12,15,24]. Furthermore, our observations suggest that a less specific Notch inhibitor will be more effective for targeting cancer cells and the tumor microenvironment, albeit with higher toxicity profile. However, only further clinical testing can ascertain this supposition.

While none of the Notch receptors have been shown to be useful as biomarkers, our *in vitro *and *in vivo *data provide evidence that the Notch pathway is oncogenic. Targeting this pathway genetically or with small molecules such as γ-secretase inhibitors may reduce tumor phenotype and represent a viable option for the treatment of patients with pancreas cancer. Because of the redundancy in oncogenic signals, targeting multiple Notch pathways will likely improve clinical outcomes. Similar to Notch, the PI3K/AKT/mTOR signaling pathway mediates key cellular processes, including cell growth, proliferation, and survival [25]. Furthermore, Akt is found to be activated in 59% of tumors. Our findings demonstrate that Notch modulates Akt, supporting a crosstalk between the pathways. While the mechanisms for this crosstalk needs further elucidation, our data suggest that one mechanism involves the modulation of PTEN phosphorylation.

PTEN is a tumor suppressor and functions as a phosphatidylinositol phosphate (PIP) phosphatase. Dephosphorylation of PI(3,4,5)P3 by PTEN prevents the phosphorylation and activation of Akt kinase [26]. Earlier studies suggest that, while phosphorylation of PTEN at the C2 domain enhances PTEN stabilization, it also promotes a closed conformation, inhibiting PTEN activity [16,27]. Conversely, in inflammatory cells, Rock1 was found to bind to PTEN and is essential for PTEN phosphorylation and activation [20]. Bone marrow cells from mice lacking functional Rock1 showed loss of PTEN activity and increased Akt activation [17]. Thus, similar to many complex biological systems, the phenotypic outcome of PTEN and RhoA/Rock pathways activation is highly context dependent.

In our system, we observed no difference in Rock1 expression with GSI, but RhoA expression was enhanced. RhoA is a member of the Rho family of small GTPases. It is required for Rock1 activation [20]. The Notch-dependent increase in PTEN phosphorylation is inhibited by Rock1 inhibitor, suggesting that Notch regulates PTEN through the RhoA/Rock1 pathway. Our study is the first to show that Notch regulates the phosphorylation of PTEN through the RhoA pathway in pancreas cancer.

We have demonstrated that the Notch pathway plays an important role in pancreas cancer. Furthermore, our findings suggest thst a cooperative relationship between the Notch pathway and the Akt/mTOR pathway may exist and this interaction is mediated by the Rho GTPase pathway. Similar to Notch, other studies have indicated a contradictory role of Rho proteins in cancer, suggesting that its role is highly context dependent. However, from the treatment perspective, Notch can be considered a target for intervention, since the inhibition of this pathway mitigates the malignant phenotype. Moreover, due to the crosstalk with the mTOR pathway, combination treatment may improve therapeutic outcome.

## Methods

### Cell lines, Constructs and Inhibitors

Human pancreas cancer cell lines Panc-1, HRAF-II and BxPC3 were obtained from American Type Culture Collection (ATCC). Murine pancreas cancer cell lines K399, K389, K375, K162, K152, and K518 were developed *ex vivo *from tumors of mice overexpressing K-rasG12D and TGF-β knockout, and were obtained from Dr. H. Moses [13]. The formulation and the *in vivo *dosing schedule of γ-secretase inhibitor MRK003 were provided by Merck Co., Inc, and were described previously [28]. The mTOR inhibitor rapamycin and the Rock1 inhibitor Y27632 were obtained from Sigma-Aldrich and CalBiochem, respectively. The γ-secretase inhibitor DAPT (N-[N-(3,5-Difluorophenacetyl)-L-alanyl]-S-phenylglycine t-butyl ester), was also obtained from Sigma-Aldrich. The dominant-negative Notch3 (DN) and VC (control) constructs were transfected into BxPC3 and selected with G418, as previously described [12]. Notch3 siRNA3 sequences were also described previously [14].

### TMA Construction, TMA Slide Preparation

De-identified tumor and adjacent normal tissues were obtained under an IRB-approved protocol at Vanderbilt University Medical Center. Before constructing a TMA block, serial 5-μm sections were cut from each donor block. One of these sections was stained with H&E for marking morphologically representative areas of the tumor. Using a Beecher Instruments Tissue Arrayer (Silver Springs, MD), tissue cylinders with a diameter of 0.6 mm were punched from the four targeted areas in each donor block and deposited into a 9 × 14 (~126 cores) TMA block, which contained 76 cores of adenoma tissue and 50 cores of adjacent, non-malignant tissue as controls. The TMA blocks were warmed to 36°C for 30 minutes, and multiple serial 5 μm sections were cut and placed on charged slides.

### Antibodies

The Notch3 antibody 1E4 (a gift from Dr. Anne Joutel) was used for immunohistochemistry, and the method was described previously [29]. Jagged1 (C-20) and Notch4 (H-225) were purchased from Santa Cruz, whereas Notch1 (3608), DLL4 (HPA023392) and Notch2 (C651.6D6HN) antibodies were obtained from Cell Signaling Technology, Sigma-Aldrich, and the Developmental Studies Hybridoma Bank (DSHB), respectively. Human EGFR antibody (31G7) was purchased from Zymed. The IHC staining was scored on a composite scale of 0 to 3 by two independent observers, including one pathologist. In case of disagreement, the decision was deferred to the pathologist. The tumors that scored 2 or better were considered positive. For immunoblotting, Notch1 (3447), Notch3 (2889), phospho-Akt, total Akt, PTEN, pPTEN, RhoA, Rock1, cdc42, Bcl-xL, Bcl-2 and PARP were obtained from Cell Signaling Technology. For specific use in murine cell lines, Jagged1 (H114), Notch1 (C-20), and Notch3 (M-134) were obtained from Santa Cruz, and Notch2 (C651.6D6HN) and Notch4 were purchased from DSHB and Orbigen, respectively.

### Real-time RT-PCR

Total RNA was isolated from K399 cells using Sure Prep RNA Purification Kit (Fisher Scientific). cDNA synthesis was carried out using iScript cDNA Synthesis Kit, according to manufacturer's recommendation (Bio Rad). Primers for murine GAPDH were AATGGGGTGAGGCCGGTG (sense) and CAGAAGGGGCGGAGATGATG (antisense). Murine RhoA primers were CCATGTACCCAAAAGCGCC (sense) and CAAATGTGCCCATCGTCCTG (antisense). Experiments were performed at annealing temperature of 55°C for 39 cycles.

### Proliferation Assays, Soft Agar and Cell Death Analysis

Cells were plated into 96-well microtitre plates at 10% FCS and at 50% confluency in 200 μl DMEM. After 48 hours of treatment with inhibitors, 50 μl of MTT (3-(4,5-Dimethylthiazol-2-yl)-2,5-diphenyltetrazolium bromide) stock solution (2 mg/ml) was added to each well, and the plates were incubated for 4 hours. MTT formazan crystals were then resolubilized by adding 150 μl 100% dimethylsulfoxide (DMSO) to each well. Plates were agitated on a plate shaker for 5 min, and the absorbance at 540 nm was determined using a scanning multi-well spectrophotometer (BioRad). For soft agar assays, transfected cells were plated at a density of 5000 cells/plate using 35 mm Petri dishes and suspended in 0.4% agar containing 10% FCS RPMI and 50 μg/ml of G418 selective antibiotic over 0.8% base agar. The plates were incubated at 37°C and 5% CO2 in a humidified chamber for 14 days. Cell death was determined as follows: Cells were stably transfected with Notch3 DN or treated with MRK003 for 24 hours and were maintained in 10% FCS-RPMI or serum-free medium. Then, they were stained with propidium iodide (Calbiochem, La Jolla, CA). The percentage of dead cells was determined with a Beckman Coulter FACS Calibur Flow Cytometer.

### In Vivo Tumorigenicity

Animal experiments were performed according to the protocol approved by Vanderbilt University IACUC. Athymic 4-to 6-week-old female nude mice (nu+/nu+) were used for the tumor xenograft models. Panc1 or K399 (1 × 106 cells in the volume of 200 ml of PBS) was inoculated subcutaneously (s.c.) into the right posterior legs of nude mice. Treatment was initiated when tumors were palpable. MRK-003 (150 mg/kg) was administered orally for three consecutive days per week for 2 weeks. MRK-003 was diluted in 0.5% methylcellulose. The tumors were measured every 2 days with a caliper. Tumor Volume (TV) was calculated with the formula: TV = (Length) × (Width)2/2. Percentage tumor volume (% TV) on day X was calculated as: %TV = (tumor volume on day ×/tumor volume on day 1) × 100.

### Statistical Analyses

The size of implanted tumors at precise time points after treatment was compared with that of control groups. Unless specifically stated, statistical inference in all comparative experiments both *in vivo *and *in vitro *was obtained using unpaired, two-sided Student's t-tests. For TMA, protein expression was correlated using Pearson's correlation coefficients. For all determinations, differences were considered significant at P < 0.05.

## Competing interests

The authors declare that they have no competing interests.

## Authors' contributions

AG and KW are pathologists. KW was responsible for production of pancreas cancer TMA, collection of clinical data, and tumor grading. AG was responsible for IHC evaluations. JB was instrumental in reviewing patients' clinical data and manuscript editing. KV and BA performed the experiments. The corresponding author is responsible for the manuscript preparation and oversaw the experiments. All authors read and approved the final manuscript.
